# The effect of education on safe use of pesticides based on the health belief model

**DOI:** 10.1186/s13104-024-06797-6

**Published:** 2024-05-13

**Authors:** Habibeh Ahmadipour, Zahra Nakhei

**Affiliations:** 1 Community Medicine Department, Afzalipour School of Medicine, Social Determinants of Health Research Center, Institute for Futures Studies in Health, Kerman University of Medical Sciences, Kerman, Iran; 2 Department of Medical Education, Medical Education Development Center, Kerman University of Medical Sciences, Kerman, Iran

**Keywords:** Education, Pesticides, Health belief model, Farmers

## Abstract

**Objective:**

In agricultural activities, pesticide use is critical, but poisoning issues are one of the most important occupational hazards for farmers. Training can help protect farmers’ health from pesticide hazards. This study aimed to investigate the effect of education on farmers’ behavior in the safe use of pesticides using the health belief model.

**Methods:**

A quasi-experimental (pretest-post-test) study conducted on 84 farmers who were selected using the convenience sampling method. The data collection tool was a two-part questionnaire including demographic information and a questionnaire designed based on the constructs of the health belief model in using personal protective equipment while working with the pesticides. The instrument was completed before and two weeks after an educational intervention. Data analysis was performed using SPSS software version 26.

**Results:**

The mean age of the participants was 48.94 ± 9.14 years and 69% were male. The study showed that with increasing age, the mean score of health belief model constructs in the safe use of pesticides decreased. Female and higher-educated farmers had higher scores. After the intervention, the mean scores of health belief model constructs in the safe use of pesticides increased significantly, except perceived barriers construct which decreased significantly. Also, the frequency of protective equipment uses while working with pesticides increased significantly after the intervention and safe behaviors increased, while unsafe behaviors decreased.

**Conclusion:**

The education as an effective intervention, improves farmers’ safety attitudes and behaviors in pesticide use and it is recommended that educational programs be designed according to the characteristics of the audience.

## Introduction

Agriculture is known as one of the most challenging jobs worldwide. Farmers are in continuous contact with dangerous factors such as chemical poisons, agricultural tools, harsh climatic conditions, and even biological factors that continuously damage their health. Failure to pay attention to the factors that threaten the health of farmers can lead to a decline in the quality of their performance, a decrease in productivity in agriculture, and ultimately a negative impact on the food security of the society [1–3]. 

Farmers are exposed to serious risks of poisoning due to the widespread use of pesticides to manage and control pests and diseases. In developing countries, due to technical limitations and lack of access to personal protective equipment, increasing the possibility of encountering health problems due to improper use of pesticides indicates fundamental challenges [4, 5]. Proper planning to raise the awareness of farmers in the field of safe use of pesticides, providing technical training, and providing personal protective equipment are of fundamental importance. Actions in this field can play an effective role in reducing the level of poisoning and improving the working conditions of farmers in developing countries [6]. 

The results of various studies in developing countries regarding the use of pesticides in agricultural operations show the low knowledge and awareness of farmers [7, 8], the lack of use of personal protective equipment [6], unsafe methods in the maintenance and storage of pesticides at home and farm [9], unsafe management of pesticide waste such as empty containers and pesticide residues [10], indiscriminate use and relatively little knowledge about safety labels on pesticide containers [11]. In Iran, insufficient knowledge of farmers [12, 13] and inattention to the use of personal protective equipment [6] in the use of pesticides have been mentioned in various researches.

Lack of knowledge in the field of safe use of pesticides is one of the key factors that lead to the occurrence of unsafe behaviors of farmers when using these chemicals. Therefore, emphasis on improving the knowledge and training of farmers regarding the safe use of pesticides and promoting optimal management practices can be effective in reducing risks and ensuring safety [14]. 

In the field of health education, theories and models have been introduced as analytical frameworks to understand the effective factors in health behaviors. One of these models is the health belief model (HBM). According to the HBM, to adopt preventive actions such as using personal protective equipment, people must first feel the risk of contracting diseases related to pesticides (perceived sensitivity). Then, they understand the depth of this risk and the seriousness of its various complications in their physical, psychological, social and economic dimensions (perceived severity), with the positive signs they receive from their surroundings or internal environment (guidelines for action), useful and believe in the applicability of protective behaviors to prevent diseases (perceived benefits) and consider the inhibiting factors (perceived obstacles) from taking action to be less expensive than its benefits, so that finally, to perform protective behaviors (use of personal protective equipment) when using pesticides [15].

According to the above-mentioned issues and since one of the main occupations in the Anbarabad region is agriculture, it is necessary to investigate the way of using pesticides among the farmers of this region and to investigate the changes in these behaviors after the educational intervention. Therefore, the current study aimed to investigate the impact of education on the behavior of farmers in Anbarabad city in the safe use of pesticides based on the health belief model in 2023.

## Materials and methods

### Study design and setting

A semi-experimental (pre-test - post-test) study was conducted in Anbarabad (Kerman province, Iran) between October and December 2023.

### Participants

84 farmers were included in the study using the convenience sampling method.The sample size in this study was calculated based on Chen et al.‘s paper [16] for the sample size determination in paired samples, taking into account the confidence coefficient of 95% the power of 80%, and the effect size of 0.31.

The inclusion criteria were being farmers and residents of Anbarabad and informed verbal consent to participate and the Exclusion criteria were the absence of more than two sessions in training sessions and failure to answer more than 10% of the questions.

### Ethical consideration

This study was approved by the ethical approval Code: IRKMUREC.1402.031 of the Ethics Committee of Kerman University of Medical Sciences. Informed verbal consent to participate in the study was obtained from all participants. The questionnaires were completed voluntarily and anonymously and with only a specified code. People were assured about the confidentiality of information and its use for research purposes.

### Data collection

The data collection tool was a two-part questionnaire, the first part of which includes demographic information (age, gender, education level), and a questionnaire designed by Seydi and colleagues based on the components related to the health belief model in the field of farmers’ safety behavior in the use of personal protective equipment in working with pesticides.

This questionnaire contains 27 items based on a 5-point Likert scale (1: completely disagree and 5: completely agree) in the form of six components perceived sensitivity, perceived severity, perceived benefits, perceived barriers, self-efficacy, and action guidelines. In addition, 6 questions measure the state of using personal equipment in working with pesticides based on a 5-level Likert scale (never: 1, rarely: 2, sometimes: 3, usually: 4, and always: 5). Content validity, construct validity (convergent and discriminant) and reliability of the questionnaire were confirmed. Convergent validity was assessed with three criteria: standardized factor loadings, average variance extracted, and composite reliability. The discriminant validity was also confirmed by using two indicators, mean squared common variance and maximum squared common variance [17]. To assess the farmers’ behavior in using personal protective equipment (in the numerical range between zero and one), the following formula was used:

Performance: (actual value-maximum value)/ (maximum value-minimum value).

After calculating the numerical value of performance, farmers’ safety behavior was classified into five levels; unsafe behavior (0.00-0.20), potentially unsafe behavior (0.21–0.40), average behavior (0.41–0.60), potentially safe behavior (0.61–0.80) and safe behavior (0.81-1.00) [18].

In the first stage, the questionnaires were completed when the farmers came for training sessions. Then, the educational content related to safe behaviors in the safe use of pesticides is presented during 4 sessions for 2 h and one day a week in the health education hall of Anbarabad Health Center. The questionnaire was administered again two weeks after the end of the training. In cases where the farmer was illiterate, the questionnaires were completed by the researcher through interviews. The time to complete the questionnaire is 15 min.

### Data analysis

Statistical analysis of the data is performed using SPSS software version 26 at two descriptive and inferential levels. Frequency, percentage, mean, and standard deviation were used to describe the data and paired t-test and McNemar test were used for comparisons.

## Results

A total of 84 farmers were studied with a mean age of 48.94 ± 9.14 years and male majority and most of them had a under diploma degree(Table 1).

Table 2 shows the comparison of the mean score of the constructs of the health belief model in the safe use of pesticides by the farmers of Anbarabad before and after the educational intervention.

**Table 1:** The characteristics of the participating farmers

Variables	Categories	Frequency	Percent
Gender	Male	58	69.0
	Female	26	31.0
Educational level	Under diploma	51	60.7
	Diploma	24	28.6
	Academic	9	10.7

**Table 2:** The comparison of the mean score of the constructs of the health belief model in the safe use of pesticides in the farmers of Anbarabad before and after the educational intervention

		Mean	Std. Deviation	*P*
Perceived sensitivity	Before	14.46	3.15	< 0.001
	After	17.64	2.52	
Perceived severity	Before	14.73	3.06	< 0.001
	After	17.61	2.49	
Perceived benefits	Before	18.63	3.52	< 0.001
	After	21.98	2.99	
Perceived barriers	Before	21.20	3.90	< 0.001
	After	15.02	3.39	
Self-efficacy	Before	14.44	2.86	< 0.001
	After	17.57	2.26	
Cues to action	Before	14.64	2.59	< 0.001
	After	17.61	2.16	
Use of personal protective equipment	Before	13.27	5.33	< 0.001
	After	22.70	4.07	

Significance level is less than 0.05

Based on this table, the mean of all the constructs of the health belief model in the safe use of pesticides has increased significantly after the educational intervention (*P* < 0.001) while the mean score of perceived barriers has significantly decreased after the intervention(*P* < 0.001). Also, the score of using personal equipment has increased significantly after the intervention (*P* < 0.001).

Tables 3 and 4 show the comparison of the mean score of the constructs of the health belief model in the safe use of pesticides in the farmers of Anbarabad before and after the educational intervention based on the farmers’ characteristics. Similar changes were observed in subgroups with general analysis, and in some constructs of the Health Belief Model, the mean scores were significantly different based on gender and educational level.

**Table 3:** The comparison of the mean score of the constructs of the health belief model in the safe use of pesticides in the farmers of Anbarabad before and after the educational intervention based on gender

		Gender		*P*
		Male	Female	
Perceived sensitivity	Before	13.94(0.40)	15.61(0.60)	0.024
	After	17.27(0.32)	18.46(0.48)	0.046
P		< 0.001	< 0.001	
Perceived severity	Before	14.22(0.39)	15.88(0.58)	0.021
	After	17.31(0.32)	18.30(0.48)	0.090
P		< 0.001	< 0.001	
Perceived benefits	Before	18.10(0.48)	19.80(0.67)	0.040
	After	21.70(0.39)	22.61(0.58)	0.200
P		< 0.001	< 0.001	
Perceived barriers	Before	21.29(0.51)	21.00(0.77)	0.753
	After	15.36(0.44)	14.26(0.66)	0.174
P		< 0.001	< 0.001	
Self-efficacy	Before	14.53(0.37)	14.23(0.56)	0.656
	After	17.48(0.29)	17.76(0.44)	0.595
P		< 0.001	< 0.001	
Cues to action	Before	14.60(0.34)	14.73(0.51)	0.837
	After	17.56(0.28)	17.739(0.42)	0.754
P		< 0.001	< 0.001	
Use of personal protective equipment	Before	0.28(0.03)	0.35(0.04)	0.188
	After	0.67(0.02)	0.73(0.03)	0.137
P		< 0.001	< 0.001	

Significance level is less than 0.05

**Table 4:** The comparison of the mean score of the constructs of the health belief model in the safe use of pesticides in the farmers of Anbarabad before and after the educational intervention based on the educational level

		Educational level			*P*
		Under diploma	Diploma	Academic	
Perceived sensitivity	Before	13.76(0.41)	14.87(0.60)	17.33(0.99)	0.004
	After	17.29(0.33)	17.50(0.49)	20.00(0.80)	0.010
P		< 0.001	< 0.001	0.004	
Perceived severity	Before	14.05(0.40)	15.04(0.58)	17.77(0.96)	0.002
	After	17.33(0.33)	17.33(0.48)	20.00(0.79)	0.009
P		< 0.001	< 0.001	0.014	
Perceived benefits	After	17.88(0.46)	18.87(0.67)	22.22(1.10)	0.002
	Before	21.54(0.40)	21.87(0.58)	24.77(0.95)	0.010
P		< 0.001	< 0.001	0.015	
Perceived barriers	Before	20.98(0.55)	21.87(0.80)	20.66(1.31)	0.599
	After	15.64(0.46)	14.66(0.67)	12.44(1.09)	0.026
P		< 0.001	< 0.001	< 0.001	
Self-efficacy	After	14.09(0.38)	14.08(0.55)	17.33(0.90)	0.005
	Before	17.49(0.31)	17.16(0.45)	19.11(0.74)	0.081
P		< 0.001	< 0.001	0.094	
Cues to action	Before	14.15(0.34)	14.70(0.50)	17.22(0.81)	0.004
	After	17.49(0.29)	17.33(0.43)	19.11(0.70)	0.087
P		< 0.001	< 0.001	0.06	
Use of personal protective equipment	After	0.23(0.02)	0.33(0.04)	0.58(0.06)	0.001
	Before	0.67(0.02)	0.70(0.03)	0.81(0.05)	0.062
P		< 0.001	< 0.001	0.004	

Significance level is less than 0.05

Table 5 shows the comparison of the frequency of use of personal equipment while working with pesticides before and after educational intervention. Based on this table, after educational intervention, safe behaviors in pesticide use in farmers increased and unsafe behaviors decreased.

**Table 5:** The comparison of the frequency of use of personal equipment while working with pesticides before and after educational intervention

Use of personal protective equipment		*N* (%)	*P*
Unsafe behavior	Before	28(33.3)	0.018*
	After	3(3.6)	
Potentially unsafe behavior	Before	31(36.9)	
	After	2(2.4)	
Intermediate behavior	Before	15(17.9)	
	After	10(11.90	
Potentially safe behavior	Before	9(10.7)	
	After	48(57.1)	
Safe behavior	Before	1(1.2)	
	After	21(25.0)	

Significance level is less than 0.05

## Discussion

The current study aimed to investigate the effect of education on the behavior of farmers in the safe use of pesticides based on the health belief model. According to our results, only about 12% of farmers had safe and potentially safe behavior in using personal equipment at the time of pesticide application. The results of research on the farmers in Andimeshk city showed an unfavorable status of the farmers’ behavior in pesticide use. In their conclusion, having appropriate knowledge and safe behavior in different stages of working with pesticides such as purchase, storage, composition, spraying and proper disposal of its waste has been emphasized to prevent health-threatening risks [19]. However, a study in China revealed that even with adequate knowledge about pesticides, many farmers did not use appropriate protective equipment when using pesticides [20]. Another study in Iran also found that a small number of farmers (8.9%) had safe behaviors in using personal protective equipment when working with pesticides. In addition, although farmers attach great importance to all components of safety behavior, in practice, due to various barriers such as lack of access and financial problems, they did not show any interest in safety behaviors when working with pesticides [21].

Our study showed that there is an inverse and significant correlation between age and the mean scores of the perceived sensitivity, perceived severity, perceived benefits, and also the mean score of the use of personal equipment. In this way, the average of these structures decreases with increasing age. Also, the mean scores of the perceived sensitivity, perceived severity, and perceived benefits were significantly higher in females than male farmers. In addition, the mean scores of all constructs of the health belief model in the safe use of pesticides were significantly higher among farmers with a university education than others, except for the perceived barriers, which did not have a statistically significant difference based on the level of education.

In several researches, the demographic characteristics, age, agricultural experience, level of education, farm size, yield, average income and membership in agricultural organizations have been effective in the perception of the health risks of farmers regarding the use of chemical pesticides [22–24].In the findings of Janmaimol and Vatenb, the influence of socio-demographic factors (age, gender, education, socio-economic status, and membership in organizations), previous experiences, attitude, mental norm, and the ability to control risk was also confirmed on the perception of health risk [25]. The results of Yaquti et al.‘s study showed that the level of education predicts the average awareness of personal protective equipment [26]. Therefore, it seems necessary to consider some demographic characteristics to raise awareness about the use of personal protective equipment while working with pesticides.

The current study showed that in overall and subgroup analyses, the mean of all the constructs of the health belief model in the safe use of pesticides has increased significantly after the educational intervention while the mean score of perceived barriers has significantly decreased after the intervention. Also, the score of using personal equipment has increased significantly after the intervention.

Various studies conducted in Iran using the HBM among farmers indicated a cognitive-informational weakness (low awareness of the side effects of using pesticides, little information about the principles of pest control and the benefits of organic farming), the weakness of educational-extension services (information Weakness and difficulty in accessing information, lack of relevant training courses, and limited access to the number of experts.(27,28) Therefore, the need for educational programs in this field is strongly felt.

## Strengths and limitations

Most of the studies conducted in Iran have examined the status of using personal protective equipment in the use of pesticides cross-sectionally using the health belief measure. The strength of the current study is its quasi-experimental nature, which examines the effect of an educational intervention on improving the safety behavior among farmers while working with pesticides. On the other hand, the present study was conducted on a limited population and in limited geographic area, so one should be careful in generalizing the results.

## Conclusion

The results of the present study showed that training as an effective intervention has improved farmers’ attitudes and safe behaviors in the use of pesticides, and it is recommended that training programs develop according to the characteristics of the target population.

## Data Availability

All data of the current paper are included in this published version.
